# The Impact of Dermal Characteristics on Low-Level Laser Power Measurement in Postmortem Zoological Species

**DOI:** 10.1155/2024/8066943

**Published:** 2024-08-26

**Authors:** Faith Ramsey, Michelle Borsdorf, John Ladner, Anne White, Tara M. Harrison

**Affiliations:** ^1^ Department of Clinical Sciences North Carolina State University College of Veterinary Medicine, Raleigh 27607, NC, USA; ^2^ Department of Clinical Veterinary Medicine University of Illinois at Urbana-Champaign College of Veterinary Medicine, Urbana 61802, IL, USA; ^3^ Canadian Food Inspection Agency, Dartmouth B3B 1Y9, NS, Canada

## Abstract

Photobiomodulation therapy, also termed as low-level laser therapy, is commonly used as an adjunctive therapy for various medical conditions in veterinary practice. The ACTIVet PRO low-level laser has been used for treatment of various nondomestic species, yet the effects of dermal attributes such as pigment, feathers, or scales have not been evaluated. The effects of low-level laser therapy with the ACTIVet PRO have been investigated in laboratory animals, including a study in rats that evaluated the passage of laser light through the skin in postmortem samples. The objective of this study was to measure the power of a low-level laser (ACTIVet PRO) after penetration through dermal tissue (∼1 mm thickness) in a variety of postmortem animal tissue. This study sought to determine the impact of fur, feathers, scales, and different pigments on the ability of the laser to penetrate. Frozen and thawed skin tissue samples from various species were placed inside a light restricted laser box and exposed to a preprogrammed laser level from a Multi Radiance ACTIVet PRO photobiomodulation (PBM) device, with a power meter to measure the light penetration through the tissue samples. Light penetration measurements via power output measurements (mW) were recorded at 7 time points (range, 1–150 sec). A Friedman test was performed to evaluate the difference of the mean tissue penetration by each species at each time point. Lighter colored specimens had higher power readings than darker colored or pigmented samples, and feathers appeared to inhibit the laser, showing minimal to no power readings on bird skin covered in covert and down feathers. There was statistically significant mean tissue penetration for all time points between the rabbit and green sea turtle (*p*=0.0046), the red-tailed hawk and green iguana (*p*=0.0046), and the red-tailed hawk and green sea turtle (*p*=0.000034). Overall findings found that certain skin coverings, such as feathers, appear to inhibit passage of laser light through tissue to the photo meter. Darker pigmented areas of tissue appeared to absorb the laser light, which also did not allow light passage through the tissue to the photo meter. All of this illustrates that there are differences in tissue penetration between different animal species, at least in postmortem tissues. This could necessitate adjustment of machine settings for therapeutic effect in different species, though further studies would be warranted to determine the extent to which this would be necessary. Additional studies evaluating biologically active tissues would be needed as a next step, as photobiomodulation has an effect at the cellular level and the exact amount of medical benefit is not measurable in skin samples that are separate from a living organism.

## 1. Introduction

The acronym LASER stands for “light amplification by stimulated emission of radiation” [1], which is now commonly used as a noun. In veterinary medicine, lasers are commonly used for a variety of surgical and therapeutic procedures. Photobiomodulation therapy (PBMT) is a common form of laser use in the veterinary field, which “results in beneficial therapeutic outcomes including but limited to the alleviation of pain or inflammation, immunomodulation, and promotion of wound healing and tissue regeneration” [2–5].

The ability of a laser to penetrate tissue depends on the wavelength of the laser [6]. Wavelengths that minimize light scattering and reflection at the surface of the tissues and absorption by chromatophores are imperative for optimal tissue penetration [7]. The targeting of deeper cells for therapy requires lasers to emit wavelengths in the 800–1000 nm range, which is considered the “therapeutic” or “optical” window for PBMT as this range allows for chromophores within tissue to absorb light [8]. Laser light also emits three wavelengths that allow it to penetrate through tissues and down to the cellular level [8]. These include monochromatic (single-color wavelength), collimated (nondivergent) and coherent (in-phase wavelengths) light [9]. The monochromatic wavelength allows for photon emissions from one set anatomic energy level. The collimated wavelength achieves a narrow beam, and the coherent wavelength allows for photons traveling in phase with each other [8].

There have not been many studies performed that would determine what effect fur, feathers, scales, and skin pigment have on the penetration of PBMT light into tissue [6]. Additionally, there are very few studies demonstrating the use of PBMT in these species [10, 11]. We believe that there are factors that could influence the efficacy of PBMT such as tissue thickness/density, location on the body, the therapy is being performed on, tissue type, tissue pigment and hemoglobin concentration, antemortem vs. postmortem tissue, and temperature. We hypothesized that the power measurement would be greatest in tissues that lack pigment (e.g., white fur and pale skin) and/or that lack a material that could disperse or impede the passage of the PBMT light wavelength (e.g., feathers or thick scales).

## 2. Materials and Methods

### 2.1. Animal Samples

Postmortem samples of 10 rabbits (*Oryctolagus cuniculus*), 12 guinea pigs (*Cavia porcellus*), 6 green iguanas (*Iguana iguana*), 11 American bullfrogs (*Lithobates catesbeianus*), 13 red-tailed hawks (*Buteo jamaicensis*), 2 green sea turtles (*Chelonia mydas*), 22 Eastern Box turtles (*Terrapene carolina carolina*), and 1 Kemp's Ridley sea turtle (*Lepidochelys kempii*) were used for this project (sea turtle samples collected under NC Wildlife Commision Endangered Species permit 18ST42). All samples were frozen and then thawed under refrigeration (40 degrees Fahrenheit) prior to use for this project. Dermal dissection was performed using a #11 scalpel blade with a linear incision medially, preserving the dorsal and anterior surfaces of the stifle region, and extending from the mid-femur to mid-to-distal tibia, yielding 4–6 cm of irregularly shaped (though most closely resembling rectangles). Samples were used immediately after sectioning off the postmortem specimen. Each skin sample was exposed to the laser only once due to biological transformation of the tissue. Skin color, skin thickness (measured with digital callipers), and temperature (determined by infrared thermometer (10 : 1 Infrared Temp Gun, Milwaukee Tool, Brookfield, WI 53005) on the margin of the tissue to avoid the location of the PBMT) were recorded for each sample. Samples were analyzed in the same room under standard room temperature of 70 degrees Fahrenheit.

Of the 10 rabbits, two samples were taken from 9 animals, one sample from each leg. 12 guinea pigs were used with two samples taken from each animal. For the red-tailed hawk specimens, two samples were taken from 8 of the 13 specimens. Two samples were taken from the one Kemp's Ridley sea turtle and both green sea turtles. Two samples were taken from the 11 American bullfrogs. Only one of the Eastern Box turtles had two samples. One sample from six green iguana specimens was used.

### 2.2. Instruments

An MR4 ACTIVet PRO (Multi Radiance Medical, Solon, OH 44139) low-level laser 1000 Hz (program 3, 905 nm) laser was used for this study. The laser was set to on for 5 minutes at 250 Hz prior to any sample collection.

The Optical Power Meter System (Thorlabs Instruments, Newton, NJ) was used to measure laser mean power output. The optical power meter system consisted of a PM200 console display unit with a 6 Hz sample rate and ± 1% accuracy. The S322C power meter sensor had a 4 cm^2^ aperture area with an optimal power range from 100 uW to 1.0 W. Power meter calibration was performed just prior to the first sample to ±1% and minimal variation was expected over the time period.

The software program utilized was PowerMax PC 0601A14R (Coherent, Wilsonville, OR). The specified measured wavelength was 905 nm. Each recording was logged in addition to manual notation at time intervals of 1, 10, 30, 60, 90, 120, and 150 seconds.

**Figure 1:**
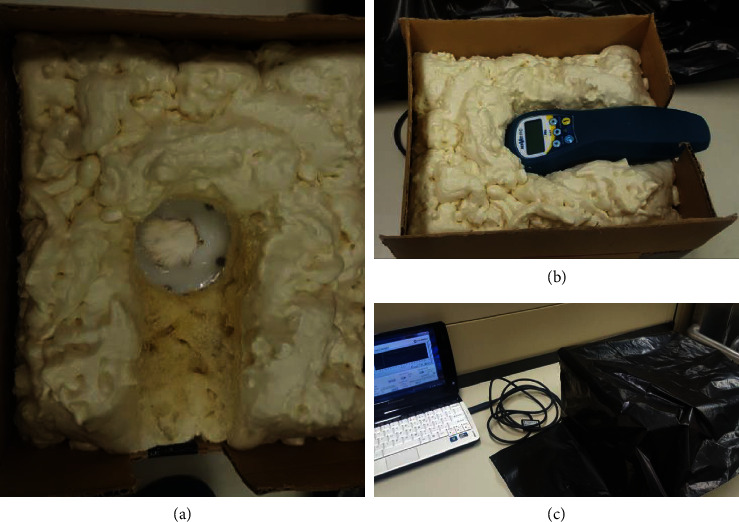
A hand-constructed stand was used to facilitate power measurements of the ACTIVet PRO photobiomodulation laser through postmortem dermal tissue of multiple zoological species. (a) A Styrofoam platform with circular cut-out illustrating a rabbit skin sample overlaying the optical power meter. (b) A cardboard box surrounding fixed spray foam holding an ACTIVet PRO photobiomodulation laser in place over a tissue sample and optical power meter. (c) The complete stand set-up with optical power meter connected to computer for data collection and covered with an opaque garbage bag to eliminate ambient fluorescent light.

A stand for the laser was constructed to maintain constant distance of the laser to the power meter (Figure 1). The power meter was secured in place with three pieces of transparent packing tape, which was housed within a circular cut-out within a Styrofoam platform that allowed exposure of the flat surface of the sensor (Figure 1(a)). The entire Styrofoam surface was then covered in saran wrap. Four external walls comprised a cardboard box surrounded the Styrofoam platform with a square cut out for the handle of the laser. Great Stuff Gaps and Cracks Insulating Spray Foam Sealant was used to specifically construct a moulded stationary rest for placement of the laser to achieve repeatable 90-degree alignment and direct contact of the convex lens of the laser head with the dermal tissue sample (Figure 1(b)). A thick black garbage bag was placed over the entire set up to eliminate possible influence of ambient fluorescent lighting (Figure 1(c)).

### 2.3. Statistics

To evaluate the difference of the mean tissue penetration by each species at each time point, the Friedman test, a nonparametric analysis of variance (ANOVA), was performed.

### 2.4. Data Collection

Prior to each measurement, the power meter was zeroed after the tissue sample was in place prior to applying the laser. Approved laser safety glasses were worn at all times while the laser was being operated. All measurements were taken in an enclosed climate-controlled room (temperature 68–72°F).

## 3. Results

**Figure 2:**
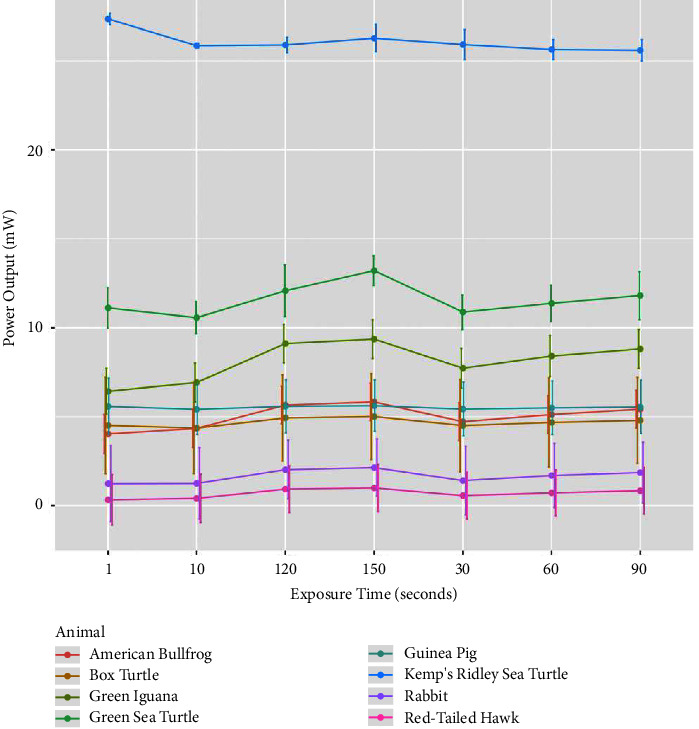
Mean tissue penetration measured via mean power output (mW) of an MR4 ACTIVet PRO photobiomodulation laser (905 nm) at predetermined exposure time points by species (American bullfrog, eastern box turtle, green iguana, green sea turtle, domestic guinea pig, Kemp's Ridley sea turtle, domestic rabbit, and red-tailed hawk) with standard error bars at each exposure time point.

The main findings of this experiment were that the color of the tissue integument appears to impact power readings as illustrated in Figure 2 for mean tissue penetration by species, as well as mean tissue thickness by species as noted in Table 1. In rabbits, their white nonpigmented skin had the highest power readings, with an average of 14.98 mW, while the grey and fawn rabbits had much lower readings (on average 0.92 mW and 0.75 mW, respectively). The black rabbit skin had almost no penetration measured (average of −0.05 mW) (Table 2). Similarly, in iguanas and bullfrogs, the lighter colored/pigmented specimens had higher power readings than the darker pigmented integument specimens, with an average reading of 22.1 mW in lighter iguana samples and 33.35 mW in lighter bullfrog samples. Conversely, the darkest iguana integument samples had an average reading of 16.42 mW and the darkest bullfrog integument samples averaged 16.32 mW. In sea turtles, Kemp's Ridley skin had higher power readings than the green sea turtle skin (average 36.88 mW and 23.16 mW, respectively). Statistically, there was a significant difference in mean tissue penetration for all time points between the rabbit and green sea turtle (*p*=0.0046), the red-tailed hawk and green iguana (*p*=0.0046), and the red-tailed hawk and green sea turtle (*p*=0.000034).

## 4. Discussion

The therapeutic outcome of PBM is the result of inducing various cellular changes, with systemic effects that include decreased inflammation via stimulation of immune cells with specific wavelengths of light, which releases inflammatory mediators [8, 12]. These are important in wound healing, pain control, and nervous system injury, among others [8]. Previous studies have not fully determined the effect fur, feathers, scales, and skin pigment have on the penetration of low-level laser light into tissues despite evaluating for clinical relevance [6, 10, 11], and thus, it is unknown what degree of tissue penetration occurred in these studies. In these studies, it was believed that a clinical difference or a factor of healing was observed, so it is presumed that the PBMT was passing through the tissues and having a biological effect [10, 11]. Although we were unable to determine biological effect directly in this study of postmortem tissues, we can compare to these publications and that with tissue penetration biological activity should be able to be observed, although the extent of biological activity and power needed would require additional research.

The bioluminescent green sheen on the inside of the skin of green sea turtles may have impacted the ability of the laser light to penetrate to the optical power meter. The same pigment trend was observed in box turtles, and although there was also a green sheen noted on some of these samples, they had a much lower average tissue penetration regardless of sample pigment/color. In red-tailed hawks, there was minimal to no power readings, other than when the feathers were compressed, in which case the power readings were higher. This observation appeared to be an actual finding, but additional studies could be performed to further clarify this. The findings in our study support our hypothesis that pigmented and darker tissues would have lower power readings. This could indicate that darker pigmented tissues do not allow the laser light to pass as readily or as deeply compared to more lightly pigmented tissues. Additionally, other attributes such as feathers and scales did negatively impact the power output readings, as predicted. The significant difference in penetration between the: rabbit and green sea turtle, the red-tailed hawk and green iguana, and the red-tailed hawk and green sea turtle further illustrated that there are differences in tissue penetration between different animal species and their postmortem tissue samples.

A potential bias of this study could be the use of postmortem samples. Tissue penetration measurements may be different in living versus nonliving tissue, such that circulating blood in living skin tissue may alter the penetration or the absorption of the laser light. Tissues were thawed prior to the study, so tissue would be less likely to cause refraction of the laser when compared to frozen samples, though it could also be possible that retained water in the tissues could have affected this study. Because the cells in these samples were dead and an enhancement of the PBM mechanisms was not possible, the actual biological activity or medical benefit of the PBMT unit could not be evaluated as a part of this study. There is increased variability of the laser itself when used over long time periods, which could produce overheating and thermal drift, although care was taken to not use the laser for long time periods during data collection sessions. Body condition of the tissues could be another potential limitation, as there are differences between muscle, fat, and water content in the tissue samples. Additionally, contusion, hemorrhage, hemoglobin content, and water can absorb more laser light which could also introduce variability between patients.

A difference was noted in the absorption of animals with extensive down and covert feathers such as the red-tailed hawk. The feathers on one hawk sample were compressed, which allowed the skin tissue to have more direct exposure to the laser light and allowed for much greater power readings compared to the noncompressed down and covert feathers. Additionally, for the samples where there was no power reading due to the darker pigment of the animal's integument or the sheen of the tissue, it is possible that instead the light was being absorbed and not passing through the integument. Another potential could be that the light was refracted, although we were unable to determine which scenario was occurring. As we were using postmortem tissues, however, we are not able to determine if this would lead to a higher PBMT effect or not.

Although some tissues did not appear to have tissue penetration measurements recorded in this study, it is still possible that they could have a metabolic benefit and that the use of PBMT should not be discouraged. Additionally, due to the differences in penetration, ideally, if the animal has feathers, a greater penetration may be achieved if the feathers are compressed or moved out of the way prior to PBMT use.

Overall, the clinical relevance of differences in tissue penetration in zoological species found in this study is difficult to determine based on using postmortem tissues. We would recommend further assessment to determine if the lower or higher level of penetration relates to therapeutic outcomes. Additional research could be done to determine if darker pigmented tissues have a greater overall positive therapeutic effect at the same setting as a lighter pigmented tissue. Further studies are also recommended in determining clinical relevance of live tissue and assess penetration levels.

## 5. Conclusions

Photobiomodulation therapy is becoming a common practice in veterinary medicine. This study was designed to evaluate the tissue penetration for PBMT light and found that lighter-pigmented tissues appear to allow for higher power output readings and thus increased laser light penetration compared to darker pigmented tissue. Also, it appears that noncompressed feathers may hinder the laser light penetration and have lower tissue penetration levels. Given the limitation of using postmortem tissues in this study, results should be assessed taking this into consideration, and further research is necessary to determine the significance of differences in laser penetration between species and skin coverings. Regardless of the readings we have reported, PBMT has been reported to have systemic effects and metabolic benefits overall; therefore, low-level laser therapy may still be beneficial, even with little or no power readings.

## Figures and Tables

**Table 1:** Mean tissue thickness (mm) by species of dermal samples that were analyzed by an MR4 ACTIVet PRO photobiomodulation laser.

Species	Mean tissue thickness (mm)
Green iguana	0.8 ± 0.17
Kemp's Ridley	0.85 ± 0.07
Green sea turtle	1.23 ± 0.05
American bullfrog	0.5 ± 0
Box turtle	1.49 ± 0.43
Rabbit	1.67 ± 0.15
Red-tailed hawk	2.19 ± 0.56
Guinea pig	1.09 ± 0.15

**Table 2:** Mean tissue penetration measured via mean power output (mW) by an MR4 ACTIVet PRO photobiomodulation laser based on species and tissue color/pigment across all time points.

Species	Tissue sample color/pigment	Mean power output (mW)
Rat	White	24.71 ± 1.36
Guinea pig	White	27.09 ± 7.2
Red-tailed hawk	White/tan/brown feathers	3.15 ± 6.1
Red-tailed hawk	^*∗∗*^without compressing feathers	1.28 ± 1.86
Kemp's Ridley sea turtle	White/tan	36.88 ± 1.01
Green sea turtle	Tan with green sheen on inside	23.16 ± 2.57
Rabbit	White	14.98 ± 3.55
Rabbit	Grey	0.92 ± 2.35
Rabbit	Fawn brown	0.75 ± 2.41
Rabbit	Black	−0.05 ± 2.75
Box turtle	Brown	56.35 ± 1.18
Box turtle	Beige pink	24.88 ± 3.56
Box turtle	Beige	20.01 ± 7.77
Box turtle	Beige/green/grey combo	17.34 ± 4.01
Box turtle	^*∗∗*^any color, green sheen noted to sample	11.91 ± 2.82
Iguana	White	22.1 ± 2.6
Iguana	Light	19.54 ± 3.5
Iguana	Light medium	16.42 ± 3.04
Bullfrog	Light brown	33.35 ± 3.58
Bullfrog	Leopard	27.68 ± 2.97
Bullfrog	Medium brown	23.9 ± 3.59
Bullfrog	Medium to dark brown	22.01 ± 4.36
Bullfrog	Dark brown	16.32 ± 3.07

^*∗∗*^indicates a special situation for this species.

## Data Availability

Data are available upon request from the corresponding author.

## References

[1] Gould G. R., Franken P. A., Sands R. H. (1959). The LASER, light amplification by stimulated emission of radiation. *The Ann Arbor Conference on Optical Pumping*.

[2] Anders J. J., Lanzafame R. J., Arany P. R. (2015). Low-level light/laser therapy versus photobiomodulation therapy. *Photomedicine and Laser Surgery*.

[3] Silveira P. C. L., da Silva L. A., Pinho C. A. (2013). Effects of low-level laser therapy (GaAs) in an animal model of muscular damage induced by trauma. *Lasers in Medical Science*.

[4] Fattahian H., Nasirian A., Mortazavi P. (2013). The role of red and infrared low level laser therapy on unmeshed full-thickness free skin autograft in rabbits: as an animal model. *Kafkas Universitesi Veteriner Fakultesi Dergisi*.

[5] Simunovic Z., Ivankovich A. D., Depolo A. (2000). Wound healing of animal and human body sport and traffic accident injuries using low-level laser therapy treatment: a randomized clinical study of seventy-four patients with control group. *Journal of Clinical Laser Medicine and Surgery*.

[6] Joensen J., Ming K. Ø., Reed R. K. (2012). Skin penetration time-profiles for continuous 810nm and superpulsed 904nm lasers in a rat model. *Photomedicine and Laser Surgery*.

[7] Jacques S. L. (2013). Optical properties of biological tissues: a review. *Physics in Medicine and Biology*.

[8] Riegel R. J., Godbold J. C. (2017). *Laser Therapy in Veterinary Medicine Photobiomodulation*.

[9] Hewitt P. G. (2001). *Conceptual Physics*.

[10] Shaver S. L., Robinson N. G., Wright B. D., Kratz G. E., Johnston M. S. (2009). A multimodal approach to management of suspected neuropathic pain in a prairie falcon (*Falco mexicanus*). *Journal of Avian Medicine and Surgery*.

[11] Archibald K. E., Harrison T. M., Troan B., Smith D., Minter L. J. (2020). Effect of multiradiance low-level laser therapy and topical silver sulfadiazine on healing characteristics of dermal wounds in marine toads (*Rhinella marina*). *Veterinary Medicine International*.

[12] Chung H., Dai T., Sharma S. K., Huang Y. Y., Carroll J. D., Hamblin M. R. (2012). The nuts and bolts of low‐level laser (light) therapy. *Annals of Biomedical Engineering*.

